# Multiple Effects, Pathways, and Potential Health Risks from Environmental Microplastic Exposure

**DOI:** 10.3390/toxics13110976

**Published:** 2025-11-13

**Authors:** Lei Su

**Affiliations:** College of Oceanography and Ecological Science, Shanghai Ocean University, Shanghai 201306, China; lsu@shou.edu.cn

## 1. Introduction

After nearly two decades of extensive research, microplastics (MPs) have been documented in virtually all ecosystems and across diverse environmental compartments [1,2]. The focus of MP research has expanded from its origins in marine pollution to the current One Health framework [3,4]. A growing body of evidence now indicates that exposure to MPs and their associated nanoplastics may pose potential risks to human health, such as increasing the incidence of cardiovascular diseases [5,6]. Consequently, determining the actual toxic effects and ecological risks of MPs represents one of the most critical research priorities. In real-world environments, MP exposure occurs through multiple pathways, and their toxic effects vary depending on their intrinsic properties—such as size, polymer type, and morphology—in addition to the exposed species [7,8]. Therefore, assessing the toxicity of MPs within authentic environmental contexts is fundamental to evaluating their potential ecological and human health risks.

In light of the limited understanding of the toxicity and mechanisms of action of MPs with different characteristics, particularly under real-world environmental conditions, we organized the following Special Issue, “Environmental Exposure to Microplastics: Effects on Animals and Human Health.” This collection comprises nine articles, including eight original research papers and one review. The articles included in this Special Issue cover a wide range of topics, from toxicological exposure experiments to field observations in environmental media and biomonitoring. They comprehensively address the toxic effects and potential risks of MPs to various organisms, including fish, birds, and mammals. Furthermore, several contributions address scenarios closely linked to human activities—such as wastewater treatment plants and the use of plastic products—thereby extending the discussion on ecological risk to the domain of human health. Collectively, this Special Issue provides valuable evidence and scientific perspectives for understanding the ecological impacts, human health risks, and exposure pathways of MPs.

## 2. An Overview of Published Articles

This Special Issue brings together nine articles that provide critical insights into the multifaceted impacts of MPs. Collectively, the research demonstrates that MPs act as carriers for co-pollutants like phthalates and pharmaceuticals, significantly enhancing their toxicity in aquatic and mammalian models. The study findings further reveal that the toxicological effects are highly dependent on polymer type, with polystyrene (PS) often exhibiting greater adverse effects than polyvinyl chloride (PVC). Fieldwork from diverse geographic regions confirms the widespread ingestion of MPs by wildlife, while innovative biomonitoring methods using bird pellets offer a non-invasive tool for tracking environmental pollution. A key finding underscores the often-overlooked pathway of human exposure through the mechanical fragmentation of plastic products during daily use. Lastly, investigations into wastewater treatment plants show that while MP removal is efficient, residual endocrine disruptors pose a persistent ecological risk. In the following sections, we systematically elaborate on these main findings.

### 2.1. Toxicological Mechanisms and Combined Exposure Effects

A cornerstone of this issue lies in elucidating the toxicological effects of MPs under environmentally relevant scenarios, particularly through combined exposure studies. The findings of studies by Zhang et al. (Contribution 3) and Wang et al. (Contribution 4), employing zebrafish models, reveal a critical synergy between MPs and co-pollutants. They demonstrate that polyethylene terephthalate (PET) and polystyrene (PS) MPs can act as carriers for Di-butyl phthalate (DBP) and methamphetamine (METH), respectively, enhancing the bioavailability and thus exacerbating the developmental toxicity and behavioral impairments induced by these chemicals. This “carrier effect” is further nuanced by the work of Xu et al. (Contribution 9), who highlight that joint toxicity is highly polymer-dependent. In their study, the authors found that while METH synergistically enhanced the toxicity of PS MPs, it antagonized the effects of polyvinyl chloride (PVC) MPs, underscoring the importance of considering plastic polymer types in risk evaluation. Complementing these aquatic toxicology studies, Gad El-Karim et al. (Contribution 1) investigated the mammalian system, showing that the plasticizer DEHP induced significant hepato-nephrotoxicity in rats through oxidative stress and inflammatory pathways, which could be effectively mitigated by the natural carotenoid lutein, pointing to potential intervention strategies.

### 2.2. Environmental Distribution and Biomonitoring

Beyond the laboratory, several studies provide vital insights into the environmental occurrence and distribution of MPs, forming the basis for ecological risk assessment. Ji et al. (Contribution 2) conducted a comprehensive survey in the Yangtze River Estuary, documenting the abundance, composition, and seasonal variations of MPs. In their work, they identified terrestrial input and hydrodynamics as key drivers of MP distribution, ultimately concluding that there is currently low ecological risk in this particular region. Extending monitoring efforts to biota, Bilal et al. (Contribution 7) and Bjedov et al. (Contribution 6) employed birds as bioindicators. Bilal et al. quantified MPs in the digestive tracts of ducks from the Panjkora River in Pakistan, confirming widespread ingestion and highlighting fragments as the dominant shape. Similarly, Bjedov et al. pioneered the non-invasive analysis of white stork pellets, successfully identifying a diverse range of anthropogenic particles and linking their composition to local industrial and waste management practices, thereby validating avian species as powerful sentinels for environmental plastic pollution.

### 2.3. Human Exposure Pathways and Risk Assessment

The pathway from environmental contamination to human exposure is a critical focus of this issue. Tong et al. (Contribution 8) directly addressed this link by investigating the fate of MPs and endocrine-disrupting chemicals (EDCs) in two wastewater treatment plants (WWTPs) in Shanghai. While WWTPs showed high removal efficiency for MPs, the persistence of EDCs in the effluent posed a non-negligible risk to receiving aquatic ecosystems. In addition, in the review by Yu et al. (Contribution 5), the authors identified a significant but often overlooked human exposure route: the mechanical fragmentation of plastic products during daily use. They argued that the release of secondary micro- and nanoplastics from items such as food packaging and textiles constitutes an acute exposure pathway, the magnitude of which may far exceed that from environmental sources, calling for an integration of product life-cycle and aging processes into risk assessment frameworks.

### 2.4. Conceptual and Methodological Advancements

Lastly, this Special Issue highlights conceptual and methodological advancements. In the review by Yu et al. (Contribution 5), the authors not only identify hidden exposure pathways but also establish a forward-looking agenda for the field. Together with the novel biomonitoring techniques demonstrated in the bird pellet and duck studies, these works push the boundaries of how we track and evaluate MP pollution, emphasizing the need for non-invasive, ethical, and comprehensive monitoring strategies that reflect real-world exposure scenarios.

## 3. Future Research Perspectives

This collection has advanced our understanding of MP toxicity and ecological risks by integrating findings from diverse case studies. Nevertheless, the toxicity of environmentally relevant MPs, particularly nanoplastics, has yet to be fully determined. Addressing these critical knowledge gaps demands technological innovation and a strategic evolution in research focus. The following key areas must be prioritized in future work to accurately assess the ecological and human health risks of MPs:

### 3.1. Inclusion of Nanoplastics in Human Health-Related Environmental Monitoring

Although MP surveys have become routine in some regions, nanoplastics have not yet been incorporated as monitoring parameters in environmentally relevant matrices closely associated with human exposure, such as drinking water. Due to their minute size, nanoplastics are more likely to penetrate human tissues and organs through environmental exposure, thereby elevating potential health risks. Moreover, once nanoplastics enter the muscular tissues of organisms, they are more prone to genuine bioaccumulation and trophic transfer, eventually reaching humans through the food web. Once technical bottlenecks in nanoplastic detection are overcome, priority should be given to establishing pollution baselines in key human-related media—including drinking water, food, and air—to support subsequent standard setting and risk management.

### 3.2. Risk Assessment of Low-Concentration and Long-Term Exposure

In real-world environments, MP exposure is generally characterized by low concentrations and long duration. However, many laboratory studies involve the employment of exposure doses significantly higher than environmental levels, which may lead to overestimation of actual risks. In addition, given the widespread use of plastic products and insufficient recycling, organisms and humans are continuously exposed to plastics through multiple pathways over extended periods. In current toxicological and medical research, long-term observational studies should be established to investigate the chronic effects of MPs, including potential intergenerational risks. There is also a need to develop more sensitive biomonitoring tools to enhance the detection of MP-related effects and enable early warning of exposure risks.

### 3.3. Considering the Impact of Aging and Weathering on Plastic Toxicity

Plastics in real-world environments are consistently subject to aging and weathering, which significantly alter their physicochemical properties. Firstly, the aging process leads to plastic fragmentation, generating secondary microplastics and thereby increasing environmental exposure concentrations. Secondly, aging modifies the surface characteristics and structure of plastics, potentially promoting the development of surface biofilms, which may enhance the likelihood of ingestion by organisms. As the structure of MPs changes during aging, plastic additives may also be gradually released. Certain additives with known endocrine-disrupting effects can form combined exposures with MPs under natural conditions. In future studies, aging and weathering processes should be regarded as critical environmental factors affecting MP behavior and toxicity, representing an essential attribute of real-world exposure scenarios.

## Data Availability

The data presented in this manuscript will be made available by the authors upon request.
